# Novel forms of *Paired*-like homeodomain transcription factor 2 (PITX2): Generation by alternative translation initiation and mRNA splicing

**DOI:** 10.1186/1471-2199-9-31

**Published:** 2008-03-28

**Authors:** Pankaj Lamba, Tord A Hjalt, Daniel J Bernard

**Affiliations:** 1 Department of Pharmacology and Therapeutics, McGill University, 3655 Promenade Sir William Osler, Montreal, Quebec H3G 1Y6, Canada; 2 Department of Experimental Medical Science, Lund University, BMC B12 Tornavagen 10, SE-22184, Lund, Sweden

## Abstract

**Background:**

Members of the *Paired*-like homeodomain transcription factor (*PITX*) gene family, particularly *PITX1 *and *PITX2*, play important roles in normal development and in differentiated cell functions. Three major isoforms of PITX2 were previously reported to be produced through both alternative mRNA splicing (*PITX2A *and *PITX2B*) and alternative promoter usage (*PITX2C*). The proteins derived from these mRNAs contain identical homeodomain and carboxyl termini. Differences in the amino-termini of the proteins may confer functional differences in some contexts.

**Results:**

Here, we report the identification of two novel PITX2 isoforms. First, we demonstrate that the *Pitx2c *mRNA generates two protein products, PITX2Cα and PITX2Cβ, via alternative translation initiation. Second, we identified a novel mRNA splice variant, *Pitx2b2*, which uses the same 5' splice donor in intron 2 as *Pitx2b *(hereafter referred to as *Pitx2b1*), but employs an alternative 3' splice acceptor, leading to an in-frame deletion of 39 base pairs relative to *Pitx2b1*. *Pitx2b2 *mRNA is expressed in both murine and human pituitary. The data show that in a murine gonadotrope cell line and adult murine pituitary what was previously thought to be PITX2B1 is actually PITX2Cβ, or perhaps PITX2B2. PITX2B1 is expressed at lower levels than previously thought. PITX2Cβ and PITX2B2 activate gonadotrope-specific gene promoter-reporters similarly to known PITX2 isoforms.

**Conclusion:**

We have identified and characterized two novel isoforms of PITX2, generated by alternative translation initiation (PITX2Cβ) and alternative mRNA splicing (PITX2B2). These proteins show similar DNA binding and *trans*-activation functions as other PITX2 isoforms *in vitro*, though their conservation across species suggests that they may play distinct, as yet unidentified, roles *in vivo*.

## Background

Homeobox genes play fundamental roles in development, including patterning and cell fate determination, and in cell-specific gene expression in adults [1]. PITX2 along with PITX1 and PITX3 form the PITX/RIEG sub-family of the *Paired*-like class of homeobox proteins [2-6]. *PITX2 *(also known as *RIEG1 *or *ARP1*) was originally cloned from a human craniofacial cDNA library and was found to be mutated in patients with Axenfeld-Rieger syndrome (ARS) [6]. ARS is an autosomal-dominant disorder of morphogenesis, characterized by malformations of the eyes, teeth, and umbilicus [6-8]. The murine gene was cloned independently by several groups and assigned various names (*Rieg*, *Ptx2, Otlx2, Brx1*) [3,5,6,8-10]. The *Pitx2/PITX2 *gene is highly conserved across species. The human and murine coding regions share 91% nucleotide (and 99.2% protein) sequence identity (PITX2A isoform, see more below) [3,6].

During murine embryonic development, *Pitx2 *is expressed in the heart, eye, pituitary, teeth, tongue, maxillary and mandibular epithelia, and in certain regions of the developing central nervous system [11-13]. Expression in the eye, dental lamina and umbilicus is consistent with a role for mutations in PITX2 in the pathogenesis of ARS. Additionally, *Pitx2 *is expressed in the lateral plate mesoderm and is expressed asymmetrically in several organs, contributing to differences in left-right patterning during embryonic development [5,14,15]. *Pitx2*-deficient mice exhibit failure of body-wall closure, arrest in organ turning, ocular defects, right pulmonary isomerism, altered cardiac position, and perturbations in early determination events in anterior pituitary gland and tooth organogenesis [12,16-18]. Mice with complete *Pitx2 *loss-of-function mutations die by embryonic day 15 due to severe heart, craniofacial and pituitary gland defects [12,19].

**Figure 1:**
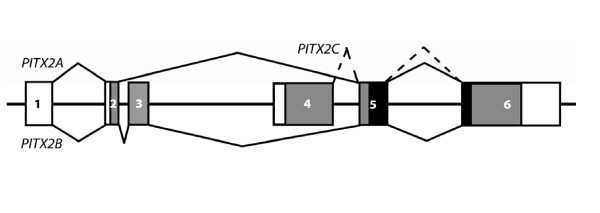
**Schematic representation of the major isoforms of PITX2 in mouse and human**. The *PITX2 *gene consists of 6 exons, which are depicted as boxes. The introns and flanking sequences are represented as lines connecting the boxes. The unfilled and filled boxes represent the non-coding and coding sequences, respectively. The homeodomain is indicated as a black region spanning exons 5 and 6. The solid line joins the exons present in *PITX2A *(top) and *PITX2B *(bottom) mRNAs; the broken line connects the exons present in the *PITX2C *mRNA. *PITX2D *is not pictured.

The *PITX2 *gene can generate different protein isoforms in humans and other mammals, the most thoroughly characterized of which are PITX2A, PITX2B, and PITX2C. Orthologs of these proteins have also been described in other vertebrates such as zebrafish, *Xenopus *and chicken [20-22]. Another mRNA isoform, *PITX2D*, was recentlycloned from a human craniofacial cDNA library [23]. The *PITX2 *gene in human and mouse is comprised of six exons. *PITX2A *and *PITX2B *mRNAs are produced through alternative splicing of the same pre-mRNA. The *PITX2A *isoform contains exons 1, 2, 5 and 6 (Fig. 1). *PITX2B *is identical to *PITX2A*, but retains an additional exon, exon 3 (138 bp), and thereby has a longer N-terminus (Fig. 1). *PITX2C *is transcribed from an alternative promoter upstream of exon 4, and therefore has a unique N-terminus, but shares its homeodomain (HD) and carboxyl terminus with the other two PITX2 isoforms (Fig. 1). In human and mouse, the PITX2A, PITX2B, and PITX2C proteins are 271, 317 and 324 amino acids in length, containing 233 common amino acids encoding the homeodomain (HD) and C-termini [3,8,11,12,19,23]. *PITX2D *derives from the same promoter as *PITX2C*, but is alternatively spliced at a cryptic 3' splice acceptor site within exon 5, and thus has a truncated HD [23]. The *PITX2D *mRNA has not been described in other species.

During development, the different PITX2 isoforms show both overlapping and distinct patterns of expression. Both the qualitative (where) and quantitative (how much) nature of their expression are critical to normal vertebrate development [17,22,24,25]. In adults, PITX2 proteins act as transcriptional regulators. *In vitro *studies show that all the three isoforms can *trans*-activate a variety of genes, including procollagen lysyl hydroxylase 1, atrial natriuretic factor, prolactin, follicle-stimulating hormone β (*Fshb*), and luteinizing hormone β (*Lhb*) [23,26-28]. Because of its truncated HD, PITX2D does not bind DNA but inhibits the transcriptional activity of other isoforms through direct physical interaction [23].

Several groups, including ours, have been investigating functional roles for PITX2 proteins in anterior pituitary cells [18,26,29-32]. *Pitx2 *is required at several different stages of pituitary development with roles in early progenitor cell formation and later lineage specification of somatotrope, lactotrope, thyrotrope and gonadotrope lineages [5,19,33,34]. PITX2 proteins are expressed in all hormone secreting cell types of the mature anterior lobe with the exception of corticotropes [5,35]. In fact, these proteins have been described as *pan*-pituitary transcriptional regulators of various hormone-encoding genes [27,30]. In the context of our recent work on PITX protein regulation of *Fshb *transcription [32], we identified two novel PITX2 isoforms. Here, we describe their characterization and examine their functions relative to the previously identified PITX2 isoforms.

## Results and Discussion

### Alternative translation initiation sites in the *Pitx2c *mRNA give rise to two proteins

**Figure 2:**
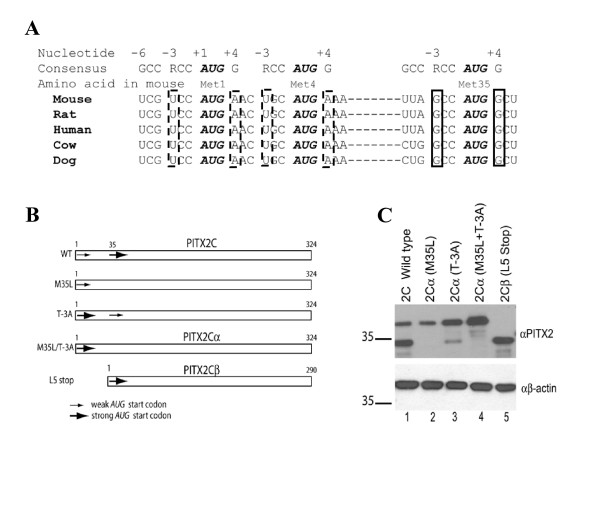
**Alternative translation initiation sites are present in *PITX2C *mRNA**. A) Comparison of the nucleotides flanking the start codon (Met1) and two other in-frame candidate start codons (Met4 and Met35) in the *Pitx2c/PITX2C *mRNAs from various mammalian species. The Kozak consensus sequence is shown at the top to facilitate the comparison. [GenBank accession nos. for the human: NM_000325, mouse: NM_001042502, rat: AJ222971, cow: NM_001097991, and dog: XM_846277]. B) Schematic representation of the expression constructs designed to study the two PITX2C isoforms. The large and small arrows represent strong and weak *AUG*^START ^codons in each cDNA sequence. C) Immunoblot analysis showing the PITX2C protein isoforms translated from the expression constructs in panel B when transfected into CHO cells. Lysates were run on a 10% Nupage^® ^Novex Bis-Tris gel with MOPS buffer.

**Figure 3:**
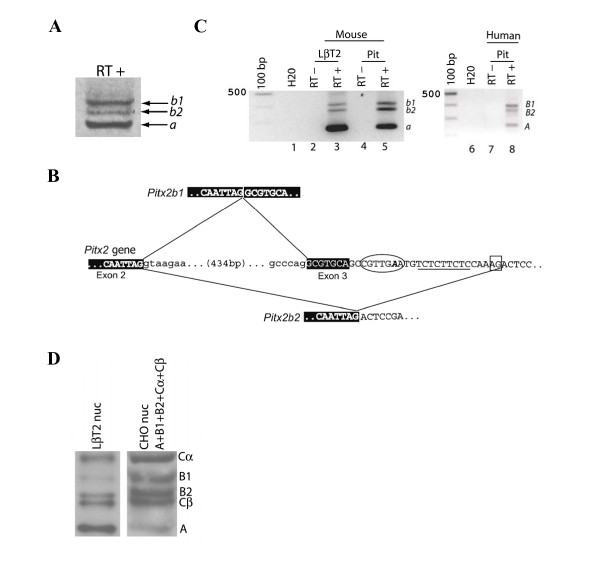
**A Novel *PITX2 *mRNA splice variant**. A) Full-length *Pitx2a, Pitx2b1*, and *Pitx2b2 *cDNAs amplified by RT-PCR from LβT2 mRNA. B) Schematic representation of the splicing events giving rise to *Pitx2b1 *(top) or *Pitx2b2 *(bottom). The 3' end of exon 2 (donor) and 5' end of exon 3 are indicated in white type with black shading. Upper and lower case letters are used for exon and intron nucleotides, respectively. The components of the lariat for the 3' alternative acceptor site are shown, with a branch point (circled, with invariant A shown as bold and italicized), followed by a poly-pyrimidine tract (underlined) and a terminal AG (boxed) at the extreme 3' end. C) RT-PCR analysis of *Pitx2a*/*PITX2A, Pitx2b1*/*PITX2B1 *and *Pitx2b2*/*PITX2B2 *mRNAs in LβT2 cells, and murine and human pituitary. D) Immunoblot analysis of PITX2 isoforms expressed in LβT2 cells (left) and combined nuclear extracts from CHO cells (right) transfected with different PITX2 isoforms run on 13.5% Tis-glycine SDS-PAGE gels. A, PITX2A; B1, PITX2B1; B2, PITX2B2; Cα, PITX2Cα; Cβ, PITX2Cβ.

During the course of our studies on transcriptional regulation of *Fshb *gene by PITX proteins in a murine gonadotrope cell line [32], we observed more than the expected three PITX2 immunoreactive proteins on western blot analyses (see Fig. 3D, left lane). We also consistently observed two PITX2 immunoreactive proteins derived from a murine PITX2C expression vector transfected into heterologous CHO cells (see Fig. 2C, lane 1). Both of these proteins bound to a PITX binding site in the murine *Fshb *promoter in southwestern blot analysis [32], suggesting that neither product was an artifact of the expression system. A review of the literature indicated that a human PITX2C expression construct similarly generated two proteins, though the nature of these proteins was not explored [23].

We therefore hypothesized that the *Pitx2c *mRNA contains alternative translation initiation codons. We examined the murine *Pitx2c *open-reading frame (ORF) (GenBank accession nos. AF048724 and NM_001042502) for downstream *AUG *codons in-frame with the canonical start site and observed two: Met4 and Met35. During translation, small ribosomal subunits enter at the 5'-end of the mRNA and migrate linearly until they encounter the first *AUG *codon (for review see [36]). In higher eukaryotes, the stop-scanning step is modulated by sequences flanking the *AUG*. In mammals, the optimal consensus sequence, GCCRCC*AUG*G (R = purine; start codon is underlined and italicized), has been identified as the most efficient context for translation initiation [36-38]. Mutations that perturb the consensus sequence, especially those that substitute a pyrimidine for a purine at position -3 (relative to the *AUG*), cause some 40S subunits to bypass the first *AUG *and continue their search for an *AUG *in more favorable context for translation initiation. This leaky ribosomal scanning is even more pronounced when the G at position +4 (the first base 3' of the *AUG*) is also altered. *AUG*^START ^codons are therefore designated strong (R^-3 ^and G^+4^) or weak based on conformity to the consensus sequence at positions -3 and +4 [36,39].

In the murine *Pitx2c *ORF, the nucleotides flanking the codons for Met1 and Met4 do not match the consensus sequence and thus provide a weak context for translation initiation (Fig. 2A, -3 and +4 are marked with dashed boxes). However, the *AUG *encoding Met35 is flanked by a strong consensus sequence due to presence of Gs at positions -3 and +4 (Fig. 2A, boxes with solid lines). We predicted that the shorter form of PITX2C (hereafter PITX2Cβ) is initiated from Met35 and the longer (canonical) form (hereafter PITX2Cα) from Met1. To evaluate this hypothesis, we introduced point mutations into the wild-type (WT) PITX2C expression construct (Fig. 2B). Briefly, we changed the downstream Met35 to Leu (M35L) to confirm that the faster mobility isoform was initiated at this amino acid and to generate a construct that expresses PITX2Cα exclusively. As predicted, the mutation eliminated PITX2Cβ (Fig. 2C, lane 2). To show that the weak initiation context of Met1 permits leaky ribosomal scanning, we replaced the thymine (T; corresponding to uracil in mRNA) at the -3 position with an adenine (T-3A). Indeed, this construct expressed PITX2Cα more and PITX2Cβ less abundantly than WT (Fig. 2C, compare lanes 1 and 3); however, some of the PITX2Cβ isoform was retained. Therefore, this single base change was not sufficient to stop leaky scanning altogether. This is likely because the +4 base was still less than optimal. Because this was the first base of codon 2, we could not change it without making a non-conservative amino acid substitution (Asn, AAC, to Asp, GAC). Because the T-3A mutation increased the abundance of PITX2Cα, it is likely that translation of this isoform occurs from Met1 and not Met4; however, this needs to be confirmed experimentally (e.g., by N-terminal sequencing of endogenous PITX2C).

To make a PITX2Cβ expression construct, we mutated Leu5 to a non-sense codon (L5Stop), thus terminating the protein translated from Met1 (or Met4) prematurely. This construct produced the PITX2Cβ isoform exclusively and at a level equal to that from the WT construct (Fig. 2C, compare lanes 1 and 5). These data further confirmed that translation initiation could occur from Met35. For downstream applications comparing functional differences between the two isoforms, we generated a construct that produced PITX2Cα exclusively and at a level comparable to that of PITX2Cβ by introducing both the M35L and T-3A mutations (Fig. 2C, lane 4).

The above results are consistent with the hypothesis that alternate initiation sites present in *Pitx2c *mRNA give rise to two, if not three, proteins by a context-dependent leaky ribosomal scanning mechanism. Because the non-consensus (Met1 and Met4) as well as consensus (Met35) nucleotides surrounding the different potential translation start sites are conserved in mammals (Fig. 2A) and in species as diverse as chicken and zebrafish (data not shown), we predict that the long and short forms of PITX2C are expressed in other species, including humans. As mentioned above, previously published, though not fully appreciated, data similarly showed that two proteins, which we predict correspond to PITX2Cα and PITX2Cβ, are produced from a human PITX2C expression construct in CHO cells [23]. The cDNA sequence for murine *Pitx2cβ *has been deposited in GenBank (acc. no. AM940439).

Similar to what we observe here, there are many examples in the literature of transcription factor mRNAs that produce short and long forms of the protein by context-dependent leaky scanning (e.g. GATA1, Pit-1, C/EBPα, EGR3, LHX3a [40-44]). As with alternative splicing and multiple promoters, this mechanism permits biologically-relevant diversity in protein synthesis from individual genes (for review, see [45]).

### A Novel *Pitx2/PITX2 *mRNA variant produced through alternative splicing

Alternative pre-mRNA splicing gives rise to *PITX2A *and *PITX2B *isoforms, with exon 3 being skipped in *PITX2A *(Fig. 1). In the course of amplifying full-length *Pitx2a *and *Pitx2b *cDNAs from the murine gonadotrope cell line, LβT2, we observed three, rather than the two expected amplicons (data not shown). Repeating the RT-PCR with DNased RNA, to remove potentially contaminating genomic DNA, showed a similar pattern (Fig. 3A). We cloned and sequenced all three products and confirmed that the top and bottom bands corresponded to *Pitx2b *(hereafter *Pitx2b1*) and *Pitx2a*, respectively. The middle band contained a novel sequence (hereafter *Pitx2b2*), highly related to *Pitx2b1*. Aligning *Pitx2b2 *with murine *Pitx2 *genomic (on chromosome 3) and *Pitx2b1 *cDNA sequences (GenBank accession nos. AC116740 and U80010) revealed that it employs the same 5' splice donor in the second intron as *Pitx2a *and *Pitx2b1*, but utilizes an alternative 3' splice acceptor within exon 3 (Fig. 3B). As a result, the first 39 bp of exon 3 are absent from this isoform. The PITX2B2 protein is predicted have 304 amino acids, lacking the first 13 residues (amino acids 16 – 28) encoded by exon 3, but otherwise being identical to PITX2B1. Analysis of the sequence 5' to this novel acceptor site indicates the presence of consensus sequences for the observed splicing event (Fig. 3B) [46,47]. Our literature search did not shed light on possible factors that might regulate the alternative splicing choices [46]. We confirmed *Pitx2b2 *expression by RT-PCR in murine pituitary, indicating that this splicing event is not an artifact restricted to the LβT2 cell line (Fig. 3C, left panel, lane 5). The cDNA sequence for murine *Pitx2b2 *has been deposited in GenBank (acc. no. AM940438).

**Figure 4:**
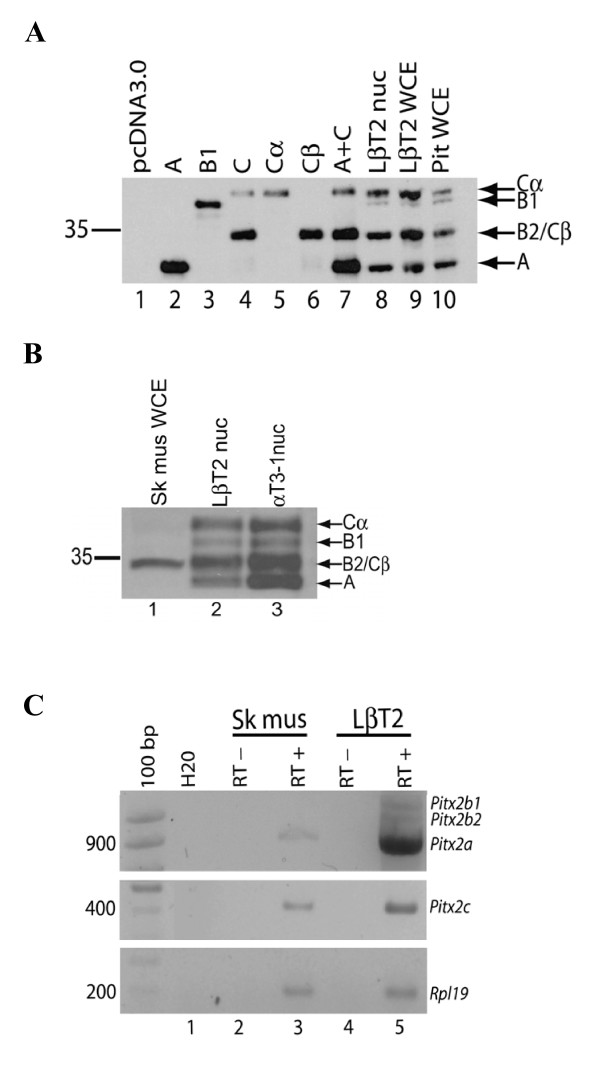
**The major PITX2 isoforms in gonadotrope cells, pituitary and skeletal muscle**. A) Immunoblot analysis of PITX2 protein expression in CHO cells transfected with different PITX2 isoforms, LβT2 cells, and murine pituitary run on a 12% Nupage^® ^Novex Bis-Tris gel with MOPS buffer. Abbreviations: pcDNA3.0, control; A, PITX2A; B1, PITX2B1; C, PITX2C; Cα, PITX2Cα; Cβ, PITX2Cβ; A+C, PITX2A and PITX2C; nuc, nuclear extract; WCE, whole cell extract. B) PITX2 immunoblot of murine skeletal muscle (Sk. mus.) WCE (lane 1), and LβT2 and αT3-1 nuclear extracts (lane 2 and 3) run on a Nupage^® ^Novex 7% Tris-acetate gel. C) RT-PCR analysis of *Pitx2a, Pitx2b1 *and *Pitx2b2 *(top) and *Pitx2c *(middle) mRNA in LβT2 cells and murine skeletal muscle (hindlimb). *Rpl19 *(bottom) was used as a loading control.

We next examined whether *PITX2B2 *mRNA is also present in human. RT-PCR of human pituitary mRNA demonstrated the expression of *PITX2A, PITX2B1*, and *PITX2B2 *(Fig. 3C, right panel, lane 8). Examination of the *PITX2 *genomic sequence on human chromosome 4 (GenBank accession nos. NM_153426 and NT_016354) confirmed that the sequence permitting alternative splicing in murine *Pitx2 *(branch point, poly-pyrimidine tract followed by a terminal AG) is also present in human *PITX2*. However, the degeneracy of splicing regulatory sequence makes it difficult to know this with certainty [47]. We were unable to identify *Pitx2b2*/*PITX2B2 *in EST database searches. This is not a major concern, as very few murine ESTs in the NCBI database correspond to *Pitx2b1*, none of which were derived from pituitary libraries. We confirmed PITX2B2 protein expression in gonadotrope cell lines, LβT2 and αT3-1 (Fig. 3D, left lane and data not shown). Comparing the migration of the endogenous LβT2 proteins (Fig. 3D, left lane) with a combination of nuclear extracts from CHO cells transfected with expression vectors for each murine PITX2 isoform (right) shows that the PITX2B2 protein migrates just above PITX2Cβ. Indeed, the predicted molecular weight of PITX2B2 (304 aa, ~34.0 KDa) is only slightly greater than that of PITX2Cβ (290 aa, ~32.2 kDa). Thus, the two proteins can be resolved only when separated on high percentage polyacrylamide gels (≥ 13.5%) (Fig. 3D). When run on lower percentage gels (e.g., as in Figs. 4A and 4B), PITX2Cβ and PITX2B2 run so closely together as to appear as one abundant protein.

PITX2 can be post-translationally modified (e.g., by phosphorylation, [7,48]); therefore, it is possible that the different isoforms we observe in LβT2 cells extracts might represent fewer isoforms than suggested, but with different states of post-translational modification. Given that the bands observed by over-expression in CHO cells show the same migration patterns and can be directly attributed to the proposed isoforms, we consider this a less likely explanation for the observed data. This does not preclude the fact that the distinct isoforms may be differentially post-translationally modified by virtue of their unique sequences. Indeed, the 34 additional amino acids in PITX2Cα include residues potentially subject to modification (i.e., Lys5, Thr17, Lys18, Ser20, Ser23, Ser24, and Cys27). Three residues in the 13 amino acids unique to PITX2B1 (i.e., Cys24, Ser 27, and Lys28) might also be modified. These possibilities should be addressed in future analyses.

### Identification of the major PITX2 isoforms expressed in LβT2 cells and adult murine pituitary

The major protein isoforms of PITX2 in mammals are generally considered to be PITX2A (271 aa), PITX2B1 (317 aa) and PITX2Cα (324 aa). In light of our results, we re-examined this hypothesis. The endogenous proteins expressed in LβT2 cells (Fig. 4A, nuclear and whole cell extracts, lanes 8 and 9) and adult murine pituitary (whole cell extract, lane 10) were compared with the different isoforms expressed in CHO cells; PITX2A (lane 2), PITX2B1 (lane 3), WT PITX2C (lane 4), PITX2Cα (M35L, lane 5), PITX2Cβ (L5Stop, lane 6), and PITX2A and WT PITX2C together (lane 7). The results suggest that: 1) the major forms in homologous cells/tissues are PITX2A, PITX2Cα and PITX2Cβ, 2) PITX2Cβ is not an artifact of our expression system and corresponds to the major immunoreactive band that migrates intermediate to PITX2A and PITX2Cα (compare lanes 6 and 8–10), and 3) endogenous PITX2B1 is expressed at relatively low levels and migrates just below PITX2Cα (compare lanes 3 and 8–10). The relative migration patterns of the different isoforms on SDS-PAGE are consistent with their predicted molecular mass: PITX2A (271 aa, ~30.3 kDa), PITX2Cβ (290 aa, ~32.2 kDa), PITX2B1 (317 aa, ~35.3 kDa) and PITX2Cα (324 aa, ~35.8 kDa). Note that the gel in this figure was not of a sufficiently high percentage to resolve PITX2Cβ and PITX2B2. In addition, based on the data in Fig. 3D, we cannot rule out the possibility that these latter two isoforms are expressed at equivalent levels in LβT2 cells and pituitary.

These data indicate that previous assessments of relative PITX2B1 protein levels were likely over-estimated. That is, three prominent bands typically detected by immunoblot using PITX2 antisera were attributed to PITX2A, PITX2B, and PITX2C [31]. Our data show that these are PITX2A, PITX2Cβ (and/or PITX2B2, co-migrating with each other), and PITX2Cα. Thus, what was previously interpreted as PITX2B1 is PITX2Cβ (and perhaps PITX2B2), and that PITX2B1 is of low abundance and is only slightly smaller than PITX2Cα (as would be predicted by the primary amino acid sequence).

These observations become particularly relevant in cellular contexts where only some of the PITX2 protein isoforms are expressed. For example, as shown in Fig. 4B, a single PITX2 immunoreactive band is observed in skeletal muscle (lane 1). PITX2 has been demonstrated to be expressed in virtually all embryonic anlagen and adult muscle groups, suggesting that the protein(s) may play some role in maturation or maintenance of muscle anlagen [49]. Previously, this protein might have been attributed to PITX2B1; however, comparing its migration to proteins in gonadotrope cell lines (Fig. 4B, lane 2 and 3) strongly suggests that it is PITX2Cβ. Indeed, we detect *Pitx2c *(Fig. 4C, middle panel, lane 3) mRNA in the skeletal muscle. Unlike the case with gonadotrope cells, we only detect one isoform on the immunoblot, raising the possibility that this protein may correspond to PITX2Cβ or PITX2B2. However, we did not detect *Pitx2b1 *or *Pitx2b2 *mRNAs in skeletal muscle and *Pitx2a *mRNA was expressed at low levels, suggesting that the upstream promoter is relatively inactive in this tissue in adult mice (Fig. 4C, upper panel, lane 3). We have observed that the PITX2Cα isoform may be more labile than the PITX2Cβ isoform (e.g., Fig. 4A, lane 4, and data not shown) and this may account for its apparent absence in skeletal muscle (Fig. 4B, lane 1). This might also be predicted based on differences between the two proteins in amino acids that confer metabolic instability.

The N-end rule, which relates the *in vivo *half-life of a protein to the identity of its N-terminal residue, predicts that PITX2Cβ would be more stable, as it has the stabilizing amino acid, alanine, at its N-terminus. In contrast, PITX2Cα has the tertiary destabilizing N-terminal residue asparagine. Our data do not definitively rule out the possibility that PITX2Cα might initiate from Met4. However, this protein would also be more labile due to the primary destabilizing N-terminal residue, lysine [50,51]. Our results lend support to the recent reclassification of alanine from a type 3 primary destabilizing residue to a stabilizing amino acid under the mammalian N-end rule [51].

### All *PITX2 *isoforms *trans*-activate gonadotrope-restricted gene promoters

PITX2 isoforms differ in their N-termini, and this contributes to variation in their *trans*-activation functions in some contexts [23]. As all PITX2 isoforms are expressed in gonadotrope cells, we compared their relative abilities to *trans*-activate gonadotrope-restricted genes: A) murine *Fshb *(m*Fshb*), B) bovine *Lhb *(b*Lhb*), C) murine gonadotropin-releasing hormone receptor (m*Gnrhr*), and D) bovine chorionic gonadotropin α subunit (b*Cga*). All of these promoters were shown previously to be stimulated by different PITX proteins in heterologous cells [27,32,52-54]. We transfected CV-1 cells (a heterologous monkey kidney cell line that was used previously in similar studies [27,32,52,53]) with the indicated reporters and PITX2 expression vectors.

**Figure 5:**
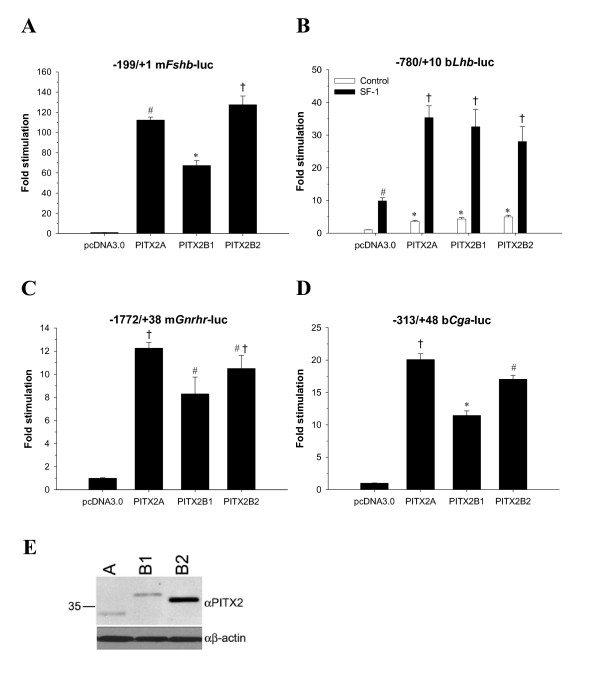
**PITX2A, PITX2B1 and PITX2B2 isoforms *trans*-activate gonadotrope-specific genes**. A, C and D) CV-1 cells seeded in 12-well plates were co-transfected with the indicated promoter-reporters (0.9 μg/well) along with empty (pcDNA3.0) or PITX2 expression vectors (0.3 μg/well). B) The *Lhb *promoter-reporter (0.9 μg/well) was co-transfected with PITX2 expression vectors plus or minus SF-1 (both expression vectors at 0.3 μg/well; DNA in all wells was balanced with pcDNA3.0). After approximately 30 h, lysates were collected for luciferase assays. Data were derived from three to five experiments performed in triplicate. Bars with different symbols differ significantly. E) Immunoblot showing relative PITX2A, B1 and B2 expression in CV-1 cells, whole cell extracts run on a 4–12% Nupage^® ^Novex Bis-Tris gel with MOPS buffer.

**Figure 6:**
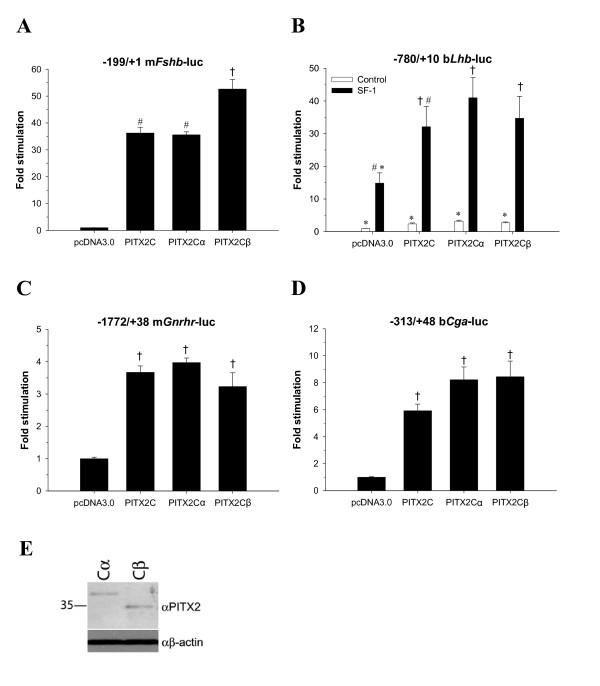
***Trans*-activation of gonadotrope-specific genes by PITX2C isoforms**. CV-1 cells were transfected as in Figure 5, except with PITX2C expression vectors [PITX2C, expressing both forms; PITX2Cα (M35L/T-3A); PITX2Cβ (L5stop)]. Immunoblot in panel E shows relative PITX2Cα and β expression in the transfected CV-1 cells (gel conditions as in Fig. 5E).

**Figure 7:**
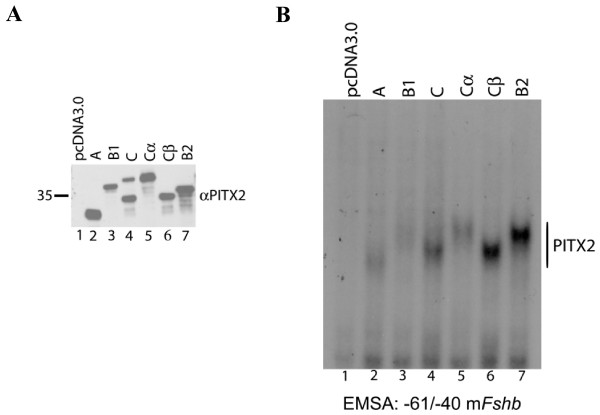
**All PITX2 isoforms bind the *Fshb *promoter**. A) Immunoblot showing PITX2 isoforms over-expressed in CHO cell extracts run on 10% Nupage^® ^Novex Bis-Tris gel with MOPS buffer. B) Gel shift assay performed with a radio-labeled probe corresponding to -61/-40 of the murine *Fshb *promoter with the same nuclear extracts used in panel A. The free probe is not pictured and lanes are numbered at the bottom.

The novel PITX2B2 and PITX2Cβ isoforms stimulated all of the gonadotrope-restricted gene promoters. PITX2B2 was more potent in stimulating the m*Fshb *promoter-reporter compared to PITX2A, which was in turn more potent than PITX2B1 (Fig. 5A). PITX2B2 was more efficiently expressed in CV-1 cells than the PITX2A or B1 isoforms (Fig. 5E) [this was not the case in CHO cells, see Fig. 7A], which might account for its greater *trans*-activation function. These data and their statistical analysis should be considered in this light. This concern does not, however, account for the differences between PITX2A and B1, which were expressed equivalently. PITX2B1 was also less effective in *trans*-activating the m*Gnrhr *and b*Cga *promoters compared to PITX2A and PITX2B2, which did not differ (Figs. 5C and 5D). We next compared the actions of PITX2Cα, PITX2Cβ, and PITX2C WT (which produces both forms). PITX2Cβ was more potent in stimulating m*Fshb *transcription compared to PITX2Cα (Fig. 6A) even though they were expressed equivalently in CV-1 cells (Fig. 6E). PITX2Cα and PITX2Cβ similarly stimulated transcription of m*Gnrhr *and *bCga *(Fig. 6C and 6D). All the PITX2 isoforms similarly synergized with SF-1 (NR5A1) to potently regulate b*Lhb *transcription (Figs. 5B and 6B). These latter results were similar to those from a previous report, which demonstrated synergism between PITX2A or PITX2B1 and SF-1 on the b*Lhb *promoter in CV-1 cells [27].

We next examined DNA binding by PITX2B2 and PITX2Cβ proteins. We demonstrated that PITX2 proteins exert their actions through a conserved *cis*-element, AAATCC, in the murine *Fshb *promoter [32]. All PITX2 isoforms bound to this element, but did so with apparently differing affinities (Fig. 7B), given their comparable levels of expression (Fig. 7A). We also conducted gel shifts with probes containing putative PITX elements from the b*Lhb*, b*Cga *and m*Gnrhr *promoters [55,56]. All of the PITX2 isoforms bound to the b*Lhb *probe [see Additional file 1(A)], but we failed to identify/confirm binding elements within the b*Cga *and m*Gnrhr *promoters under our gel shift conditions (data not shown). The PITX2 isoforms show two complexes with b*Lhb *probe [see Additional file 1(A)] similar to what was observed earlier by Cox et al. with the *Dlx2 bicoid *consensus sequence [23]. It is possible that the two complexes reflect PITX2 binding alone (monomer and dimer) or with a non-PITX partner expressed endogenously in CHO cells. As an added control, we examined the binding of the PITX2 isoforms to the recognized PITX binding element (CE3) in the rat *Pomc *promoter [4] and observed a binding pattern similar to that with the m*Fshb *probe [see Additional file 1(B)].

Collectively, the data show that all PITX2 isoforms examined here can stimulate transcription of gonadotrope-restricted promoter-reporters, though their relative effects can vary between genes. This is in agreement with results of previous studies [8,23,28,54]. In some instances (e.g., murine *Fshb*), this may be related to differences in relative binding affinities, whereas additional/alternative mechanisms, including differences in their expression levels in CV-1 cells or post-translational modifications of the proteins [7,48] may be at play in other cases.

## Conclusion

Here, we identified two novel forms of PITX2, produced by alternative mRNA splicing (PITX2B2) and alternative translation initiation (PITX2Cβ). Endogenous protein expression of both forms was confirmed in gonadotrope cell lines (PITX2B2 and PITX2Cβ) and murine pituitary and skeletal muscle (PITX2Cβ). Based on the protein and mRNA data, we suggest that the preferred splicing event is removal of exon 3, generating *Pitx2a*. When exon 3 is included, the splicing events generating *Pitx2b1 *and *Pitx2b2 *mRNAs are roughly equivalent, at least in pituitary. Our results are consistent with earlier reports demonstrating lower abundance of *Pitx2b1 *relative to *Pitx2a *mRNAs in human and murine pituitary as well in various cell lines representing different pituitary cell types [5,35]. PITX2Cβ is not only abundant, but appears to be the protein previously attributed to PITX2B1. Though, our results suggest that PITX2Cβ abundance might also be over-estimated because PITX2B2 migrates with this isoform and only higher percentage gels can faithfully resolve the two proteins.

A comparison of the *trans*-activation functions of the PITX2C isoforms showed that both stimulated various gonadotrope-specific genes in a qualitatively similar manner, though quantitatively to different extents in some circumstances. The conservation of these isoforms across species suggests that they may play distinct biological roles, perhaps independent of their roles as transcription factors, that we were unable to uncover fully here. Knock-in studies in mice introducing the mutations we describe would enable exclusive expression of the two isoforms and provide a means to assess their relative roles during development (e.g., left and right patterning) and adulthood.

## Methods

### Reagents

Dulbecco's modified Eagle medium (DMEM) with 4.5 g/L glucose, L-glutamine and sodium pyruvate was from Mediatech (Herndon, VA). Fetal bovine serum (FBS) and bovine calf serum (BCS) were bought from JRH Biosciences (Lenexa, KS). F-12/DME (1:1) medium with 1.4 g/L glucose, L-glutamine without linolenic acid was from Irvine Scientific (Santa Ana, CA). Aprotinin, leupeptin, pepstatin and PMSF were from Sigma (St. Louis, MO). Restriction endonucleases, RQ1 DNase, T4 polynucleotide kinase, deoxynucleotide triphosphates, Moloney murine leukemia virus reverse transcriptase, random primer hexamers, Taq polymerase and 5× Passive Lysis Buffer (PLB) were from Promega (Madison, WI). *Pfu*Turbo DNA polymerase was from Stratagene (La Jolla, CA). Primers and probes (Table 1) were synthesized by IDT (Coralville, IA). Poly(dI).poly(dC) and ECL-plus reagent were purchased from Amersham Biosciences (Piscataway, NJ).

**Table 1:** Primer and probe sequences

Cloning	
Pitx2a/b.forward	5' CGTACGAAGCTTATGGAGACCAATTGTCGCAAAC 3'
Pitx2c.forward	5' CGGGATCCTCCATGAACTGCATGAAAGGC 3'
Pitx2.reverse	5' AGTTGCCCACTCCGACAGTCCT 3'
Mutagenesis (sense strand)	
Pitx2c.M35L	5' CATCCCCAGGCGTTAGCCTTAGCTTCGGTCCTAGCTCCTG 3'
Pitx2c.L5stop	5' CTCCATGAACTGCATGTAAGGCCCGCTGCCCTTG 3'
Pitx2c.T-3A	5' CGATTCGGGATCCACCATGAACTGCATG 3'
Oligonucleotide probes for colony hybridization	
P1 probe	5' CCAGCAGCAAGCTGTTCCCGCGGCAGCACCCC 3'
P2 probe	5' GCCGGCAGCCGTTGAATGTCTCTTCTCC 3'
RT-PCR	
Pitx2a/b.forward	5' CGTACGAAGCTTATGGAGACCAATTGTCGCAAAC 3'
Pitx2.reverse	5' AGTTGCCCACTCCGACAGTCCT 3'
Pitx2c.forward	5' CGGGATCCTCCATGAACTGCATGAAAGGC 3'
Pitx2.HD.reverse	5' TCGGGCTTCCGTAAGGTTGGTC 3'
Rpl19.forward	5' CTGAAGGTCAAAGGGAATGTG 3
Rpl19.reverse	3' GGACAGAGTCTTGATGATCTC 3'
EMSA (sense strand)	
-61/-40 m*Fshb*	5' CACCCAGTAAATCCACAGGGTT 3'
CE3 r*Pomc*	5' ACCAGGATGCTAAGCCTCTCGTC 3'
-106/-76 b*Lhb*	5' GCCCCCGGGGAGATTAGTGTCCAGGTTACCCCACC 3'
-321/-280 m*Gnrhr*	5' TCATTAAGGCTAATTGGATGATATTATGAGTCACTTTCGACA 3'
-406/-365 m*Gnrhr*	5' TTTTAAATTGGATCGGGATTTTTAAATTACTTTTCTGTATTT 3'
-100/-68 b*Cga*	5' GGGTGGAATTTACTGTTGATCCCTGGACTTAGA 3'

Identified or known PITX binding *cis*-elements are underlined in the EMSA probes

### Animals

The indicated tissues were extracted from male wild-type CD1 mice in accordance with institutional (Rockefeller University and McGill University) and federal guidelines.

### Reporter and expression constructs

The open reading frame (ORF) of the murine *Pitx2 *isoforms: *Pitx2a*, *Pitx2b1, Pitx2c *and *Pitx2b2 *were PCR-amplified from LβT2 cell cDNA using previously described methods [57]. Reverse primer directed against the 3'UTR, *Pitx2*.reverse, was used with isoform specific forward primers (see Table 1). Amplified fragments were ligated into pcDNA3.0 (Invitrogen) and verified by DNA sequencing (Genewiz; South Plainfield, NJ). Site-directed mutagenesis was used to generate different PITX2C expression constructs from the parental wild-type vector (primers are listed in Table 1).

-199/+1 murine *Fshb*-luc was described before [32]. -780/+10 bovine *Lhb*-luc and -313/+48 bovine *Cga*-CAT were kindly provided by Dr. John Nilson (Washington State University, Pullman, WA) and -1772/+38 murine *Gnrhr*-luc by Dr. Teresa Woodruff (Northwestern University, Evanston, IL). The *HindIII-HindIII *fragment from -313/+48 b*Cga*-CAT in pSOVCAT was ligated into the same site of pGL3-Basic (Invitrogen). The proper orientation was confirmed by sequencing. All of the promoter-reporters were in pGL3-Basic, except b*Lhb*-luc, which was in pGL2-Basic.

### Cell culture, transfections and reporter assays

LβT2 and αT3-1 cells were provided by Dr. Pamela Mellon (University of California, San Diego, CA) and were cultured as described previously [32,58]. CHO and CV-1 cell lines were obtained from Dr. Patricia Morris (Population Council, New York, NY). CHO cells were cultured in F-12/DME containing 5% horse (Gibco/Life Technologies) and 2.5% BCS. CHO cells in 10-cm dishes were transfected when 70–80% confluent using Lipofectamine and 8 μg of the indicated PITX expression vector. CV-1 cells were cultured in DMEM/10% FBS. Prior to transfection, cells were seeded in 12-well plates at a density of 3 × 10^4 ^cells per well and ~36 h later were transfected using the calcium-phosphate method [59]. Briefly, DNA was diluted in 0.1× TE (pH 7.6) and CaCl_2 _[0.25 M final]. One volume of this 2× CaCl_2_-DNA solution was mixed with an equal volume of 2× HEPES-buffered saline [140 mM NaCl, 10 mM KCl, 1.5 mM Na_2_HPO_4_.2H_2_0, 50 mM HEPES (pH 7.05)]. The DNA-CaPO_4 _mixtures were incubated for 1 min at room temperature prior to adding to the cells. After 6–8 h, the medium was replaced and 24 h later the cells were washed with phosphate-buffered saline (PBS) and lysed in 1× PLB (Promega). Luciferase assays were performed on a Luminoskan Ascent luminometer (Thermo Labsystems, Franklin, MA) using standard reagents.

### Immunoblot assays

Whole cell extracts from the gonadotrope cell line, LβT2 and αT3-1, and nuclear protein extracts from CHO cells transfected with PITX2 expression vectors were prepared as previously described [32]. Protein extract from skeletal muscle (hindlimb) was prepared by grinding frozen tissue with mortar and pestle. One ml of RIPA buffer was added for every 100 mg of tissue. Further, tissue was frozen in liquid nitrogen and thawed (37 C) six times in buffer and finally centrifuged at 13,000 × g for one hour at 4 C to remove cellular debris. Immunoblots were performed using previously described methods [60]. The PITX2 antibody (P2Y4) was raised in rabbit against an epitope (DPSKKKR) N-terminal to the homeodomain, as previously described for the P2R10 antibody. This antibody recognizes all the known PITX2 protein isoforms in human, mouse and *Xenopus *[23,61], except PITX2D.

### Colony hybridization

The same PCR primers were used to generate expression vectors for PITX2A, PITX2B2, and PITX2B2. Recombinant clones were screened by colony hybridization to identify the different isoforms. Briefly, colony lifts were performed using Protran nitrocellulose membranes (Schleicher & Schuell, Keene, NH). Membranes were sequential incubated in denaturation [1.5 M NaCl, 0.5 M NaOH], neutralizing [0.5 M Tris-HCl (pH 8), 1.5 M NaCl], and wash buffers [0.2 M Tris-HCl (pH 7.5), 2× SSC]. DNA was cross-linked to the membrane using a UV cross-linker (Stratalinker 1800; La Jolla, CA). Single-stranded oligonucleotides designed to differentiate *Pitx2b1 *and *Pitx2b2 *from *Pitx2a *clones (P1) and *Pitx2b1 *from *Pitx2b2 *clones (P2) were end-labeled with ^32^P-ATP (Perkin Elmer; Waltham, MA) using T4 polynucleotide kinase (Table 1). Duplicate membranes were probed with 2 × 10^6 ^cpm/ml of the P1 or P2 probes at 42 C overnight. After washing, blots were apposed to X-ray film. Differing hybridization patterns between the two probes were used to isolate *Pitx2b1 *and *Pitx2b2 *clones. Clones were validated by restriction digest and sequencing.

### Reverse transcription-polymerase chain reaction (RT-PCR)

Total RNA from LβT2 cells and murine pituitary and skeletal muscle (hindlimb) was prepared using TRIzol (Invitrogen) following manufacturer's instructions. Human pituitary (normal adult) RNA was from BioChain Institute, Inc. (Hayward, CA). *Pitx2a, Pitx2b1 *and *Pitx2b2 *mRNA expression in LβT2 cells, murine and human pituitaries and murine skeletal muscle was examined by RT-PCR using the *Pitx2a/b*.forward and *Pitx2*HD.reverse or *Pitx2*.reverse primers (Table 1) and previously described methods [57]. These primers matched both murine and human sequences. *Pitx2c *mRNA was detected using *Pitx2c*.forward and *Pitx2*HD.reverse primers. The PCR cycling profile using Taq polymerase (Promega) consisted of an initial denaturation step at 94 C for 2 min followed by 35 – 40 cycles of amplification (94 for 30 sec, 55 for 30 sec and 72 for 30 sec). After PCR, the amplified products were subjected to electrophoresis on 2% agarose gels stained with ethidium bromide.

### Electrophoretic mobility shift assays

Gel shift experiments were performed using nuclear extracts form PITX2-transfected CHO cells as described previously [32] with the following modifications: 0.5–1 μg poly(dI).poly(dC) was used in the binding buffer as non-specific competitor. The gel shift with -106/-76 b*Lhb *probe used 0.5 μg salmon-sperm DNA. Gels were run for 2.5–3 h at 4 C.

### Statistics

Data from replicate experiments were pooled (n = 9 or 15 per treatment) for statistical analyses and are presented as fold-change from the control condition (set to 1) in each experiment. Differences between means were compared using one- or two-way analyses of variance (ANOVA), followed by Tukey post-hoc tests where appropriate (Systat 10.2, Richmond, CA). In some experiments, data were log transformed when the variances were unequal between groups. Significance was assessed relative to *p *< 0.05.

## Authors' contributions

PL contributed to the experimental design, conducted all of the experiments, performed sequence alignments and drafted the manuscript. TAH developed the PITX2 antibody and helped with preparation of the manuscript. DJB conceived of the study, participated in its design, performed the statistical analyses, and helped to draft the manuscript. All authors read and approved the final manuscript.

## Supplementary Material

Additional file 1 **PITX2 isoforms bind the PITX-binding elements in the *Lhb *and *Pomc *promoters**. Nuclear extracts used in Fig. 7 were incubated with radio-labeled -106/-76 *Lhb *(panel A) and *Pomc *(CE3) probes (panel B). PITX2 proteins formed a single complex with the CE3 probe, migrating similarly to the complexes formed with the m*Fshb *probe (Fig. 7B), and two complexes with b*Lhb *probe. These latter data are consistent with what we observed with PITX1 binding to the m*Fshb *promoter [32], suggesting that PITX2 might bind as both a monomer and dimer. Free probe is not pictured. Click here for file
