# Supplementary figures and images for: Novel forms of Paired-like homeodomain transcription factor 2 (PITX2): Generation by alternative translation initiation and mRNA splicing
Source: BMC Mol Biol. 2008 Mar 28;9:31. doi: 10.1186/1471-2199-9-31 (PMC2330153; doi:10.1186/1471-2199-9-31)

**A**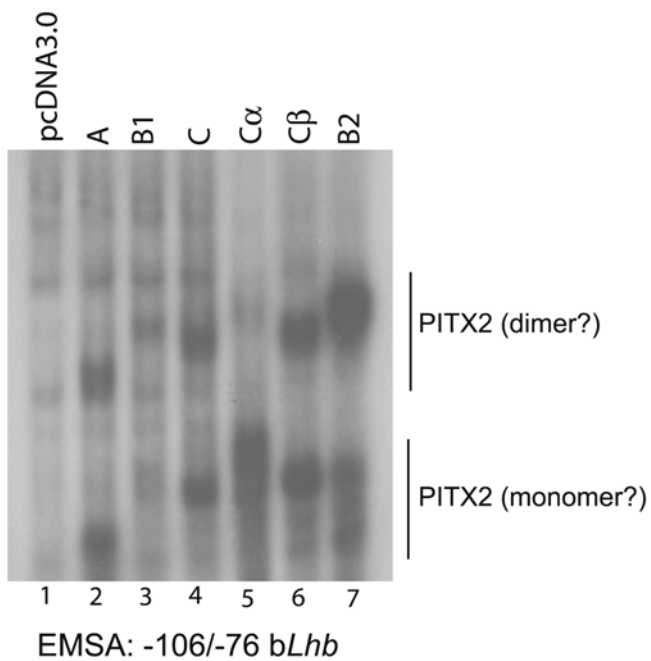**B**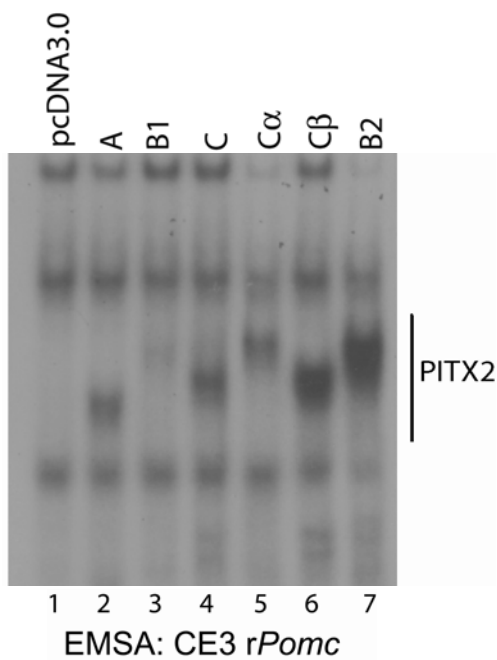

Supplement: Additional file 1 — PITX2 isoforms bind the PITX-binding elements in the Lhb and Pomc promoters. Nuclear extracts used in Fig. 7 were incubated with radio-labeled -106/-76 Lhb (panel A) and Pomc (CE3) probes (panel B). PITX2 proteins formed a single complex with the CE3 probe, migrating similarly to the complexes formed with the mFshb probe (Fig. 7B), and two complexes with bLhb probe. These latter data are consistent with what we observed with PITX1 binding to the mFshb promoter [32], suggesting that PITX2 might bind as both a monomer and dimer. Free probe is not pictured. [file 1471-2199-9-31-S1.pdf]
